# Zebrafish Adar2 Edits the Q/R Site of AMPA Receptor Subunit *gria2α* Transcript to Ensure Normal Development of Nervous System and Cranial Neural Crest Cells

**DOI:** 10.1371/journal.pone.0097133

**Published:** 2014-05-12

**Authors:** I-Chen Li, Yu-Chia Chen, Yi-Yun Wang, Bo-Wei Tzeng, Chun-Wen Ou, Yi-Yan Lau, Kan-Mai Wu, Tzu-Min Chan, Wei-Hsiang Lin, Sheng-Ping L. Hwang, Wei-Yuan Chow

**Affiliations:** 1 Institute of Molecular and Cellular Biology, National Tsing Hua University, Hsinchu, Taiwan; 2 Institute of Systems Neuroscience, National Tsing Hua University, Hsinchu, Taiwan; 3 Institute of Cellular and Organismic Biology, Academia Sinica, Taipei, Taiwan; Rutgers-Robert Wood Johnson Medical School, United States of America

## Abstract

**Background:**

Adar2 deaminates selective adenosines to inosines (A-to-I RNA editing) in the double-stranded region of nuclear transcripts. Although the functions of mouse Adar2 and its biologically most important substrate *gria2*, encoding the GluA2 subunit of AMPA (α-amino-3-hydroxy-5-methyl-4-isoxazole propionic acid) receptor, have been extensively studied, the substrates and functions of zebrafish Adar2 remain elusive.

**Methods/Principal Findings:**

Expression of Adar2 was perturbed in the *adar2* morphant (*adar2MO*), generated by antisense morpholio oligonucleotides. The Q/R editing of *gria2α* was reduced in the *adar2MO* and was enhanced by overexpression of Adar2, demonstrating an evolutionarily conserved activity between zebrafish and mammalian Adar2 in editing the Q/R site of *gria2*. To delineate the role of Q/R editing of *gria2α* in the developmental defects observed in the *adar2MO*, the Q/R editing of *gria2α* was specifically perturbed in the *gria2αQRMO*, generated by a morpholio oligonucleotide complementary to the exon complementary sequence (ECS) required for the Q/R editing. Analogous to the *adar2-*deficient and Q/R-editing deficient mice displaying identical neurological defects, the *gria2αQRMO* and *adar2MO* displayed identical developmental defects in the nervous system and cranial cartilages. Knockdown *p53* abolished apoptosis and partially suppressed the loss of spinal cord motor neurons in these morphants. However, reducing p53 activity neither replenished the brain neuronal populations nor rescued the developmental defects. The expressions of *crestin* and *sox9b* in the neural crest cells were reduced in the *adar2MO* and *gria2αQRMO*. Overexpressing the edited GluA2α^R^ in the *adar2MO* restored normal expressions of *cresting* and *sox9b*. Moreover, overexpressing the unedited GluA2α^Q^ in the wild type embryos resulted in reduction of *crestin* and *sox9b* expressions. These results argue that an elevated GluA2α^Q^ level is sufficient for generating the cranial neural crest defects observed in the *adar2MO*. Our results present a link between dysfunction of AMPA receptors and defective development of the nervous system and cranial neural crest in the zebrafish.

## Introduction

The metazoan A-to-I RNA editing is catalyzed by Adar (adenosine deaminases that act on RNA) proteins that deaminate selective adenosines to inosines in a double-stranded RNA region [1]. Altered Adar expression and RNA editing activity have been reported in human psychiatric disorders, sporadic amyotrophic lateral sclerosis (ALS), ischemia-induced neuronal death, astrocytomas, and other diseases [2]–[5]. RNA editing activity of Adar changes the protein-coding sequences and affects the biogenesis of RNAs, resulting in alternation of the protein properties and gene expression profiles. Adar proteins also affect the biogenesis of miRNA through their RNA binding abilities but independent of catalytic activity [3], [6].

Adar2 and Adar1 are two vertebrate Adar proteins that deaminate A on double-stranded RNA regions. Some RNA target sites are edited by both mammalian ADAR1 and ADAR2, but some sites are preferentially edited by either ADAR1 or ADAR2 [7]. The Q/R site of mammalian α-amino-3-hydroxy-5-methyl-4-isoxazole propionic acid receptor (AMPAR) GluA2 subunit transcript, *gria2* (*GluR-B*/*GluR2*), is preferentially edited by Adar2 [8]. The *gria2* is fully edited at the Q/R site throughout mouse development. The edited R form (GluA2^R^) subunit plays a dominant role in reducing the Ca^2+^ entry of GluA2^R^-containing AMPARs [9]. Mice with a Q/R editing-deficient allele of *gria2* (*gria2^+/ΔECS^*/*GluR-B^+/ΔECS^*), lacking the exon complementary sequence (ECS) absolutely required for RNA editing, are epileptic and die within 2 weeks of birth [10]. The phenotype of *adar2*-deficient (*adar2^−/−^*) mouse resembles that of the *gria2^+/ΔECS^* mouse and the abnormalities are rescued by replacement of the chromosomal *gria2^Q^* with *gria2^R^*, demonstrating that failing to edit *gria2* at the Q/R site is responsible for the abnormalities of *adar2*-deficient mouse [8]. *Drosophila* lacking the *adar2* homolog displays age-dependent neurological and behavior defects but is morphologically normal with normal lifespan under optimal conditions [11]. Mice defective in *adar1* are embryonic lethal, display defective hematopoiesis and widespread apoptosis in tissues expressing high levels of *adar1* [7], [12].

Zebrafish homologues of mammalian *adar* have been identified [13], [14]. A-to-I editing of zebrafish *gria2α* and kainate receptor subunit *grik2α* has also been reported [15]–[17]. Interestingly, the editing of *gria2α* during early zebrafish development is incomplete [16] and the chromosomal sequence of the other *gria2* paralogue, *gria2β*, encodes an R codon at the Q/R site [15]. Moreover, both *gria2* paralogues of more derived teleost carry chromosomally encoded R codon [15]. In this study, we demonstrate an evolutionarily conserved function of zebrafish Adar2 in editing the Q/R site of *gria2α.* Reducing *adar2* expression and reducing Q/R editing of *gria2α* resulted in severe developmental defects in the nervous system and cranial cartilages. Further studies revealed that the induction of apoptosis and reduced number of spinal cord motor neurons in the morphants depended on p53, while the developmental defects in brain, lateral line neuromasts and head cartilages were p53-independent. Results of overexpressing the edited and unedited forms of GluA2α in the *adar2* morphant and wild type zebrafish embryos demonstrate that an elevation of the unedited GluA2α^Q^α level is sufficient to disturb the development of neural crest cells in zebrafish.

## Results

### Expression pattern of *adar2*

**Figure 1:**
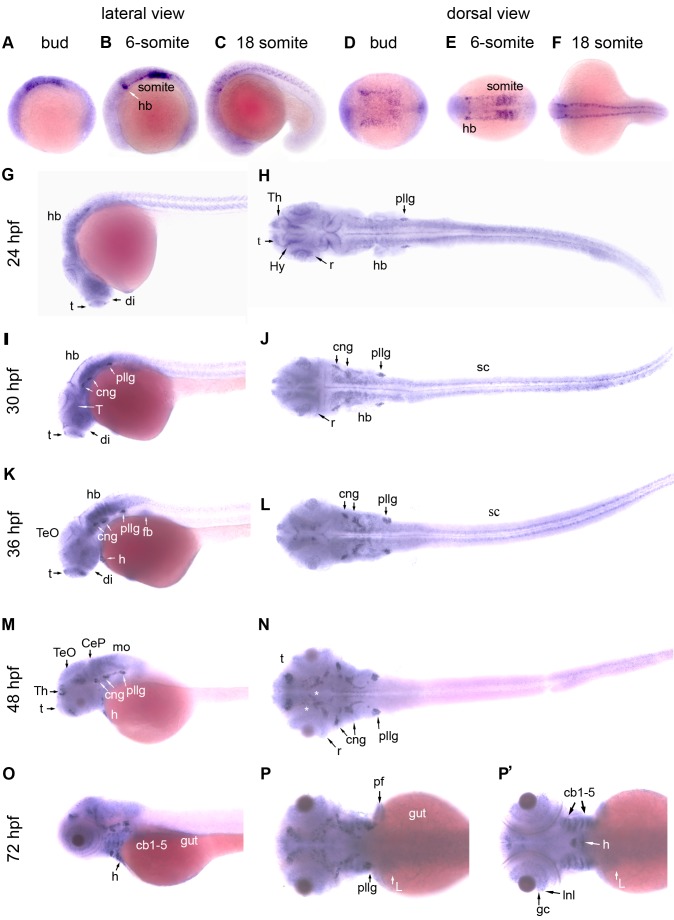
Expression patterns of zebrafish *adar2* during embryogenesis. The developmental stages are indicated on top and on the left, as hour post fertilization (hpf). (**A, B, C, G, I, K, M and O**) Lateral views and (**D, E, F, H, J, L, N, P and P’**) dorsal views of the embryos. The anterior and dorsal sides are respectively to the left and top. (**P and P’**) Images were taken from two different focuses. P’ Image is slightly deeper showing the *adar2* expression in the ventral structures. Abbreviations: cb1-5, ceratobranchials 1–5; CeP, cerebellar plate; cng, cranial ganglion; di, diencephalon; fb, fin bud; gc, retinal ganglion cells; h, heart; hb, hindbrain; Hy, hypothalamus; Inl, inner nuclear layer; L, liver; mo, medullar oblongata; pf, pectoral fin; pllg, posterior lateral line placode/ganglion; r, retina; sc, spinal cord; t, telencephalon; T, tegmentum; TeO, tectum opticm; Th, thalamus.

Quantitative RT-PCR analysis revealed a relatively high level of *adar2* transcript in the 1-cell (0 hpf) and blastrula-staged (4 hpf) embryos, indicating that maternal transcript was presented in the zebrafish embryos. The level (relative to the level of *actb1*, *β-actin*) of *adar2* transcript decreased at 10 hpf and then remained stable between 10 to 72 hpf (Fig. S1). WISH (whole-mount *in situ* hybridization) analysis revealed that *adar2* was ubiquitously expressed in the epiblast during gastrulation and early segmentation periods. Slightly higher expressions of *adar2* were detected in the neural plate of bud-stage embryos (Fig. 1A and D) as well as in the hindbrain (hb) and somites of 6-somite stage embryos (Fig. 1B and E). The expression of *adar2* became more restricted to the nervous system at later segmentation stages (Figs. 1C and F). Persistent expression of *adar2* in the forebrain (telecephalon and diencephalon), retina and cranial sensory ganglia was maintained between 24 to 72 hpf (Figs. 1G-P), while expression of *adar2* in the caudal region of CNS (hindbrain and spinal cord) decreased after 36 hpf. The expression of *adar2* in the ventral midbrain (tegmentum) became more prominent at 30 hpf (Fig. 1I). At 48-hpf, enriched expression of *adar2* was observed in discrete areas of ventral midbrain, matching the locations of cranial motor neurons (asterisks, Fig. 1N). In addition to the expression in the nervous system, *adar2* was highly expressed in the heart (Figs. 1K, M, O, and P’) and the third to seventh pharyngeal arches (cb 1–5, Fig. 1O and P’). Low levels of *adar2* expression were also detected in the fin bud/pectoral fin, liver and digestive tract (Fig. 1L, N, P and P’).

In general, the expression domains of *adar2* in the CNS and cranial sensory neurons overlapped with that of the AMPAR subunit genes, *gria1-4* [18]. However, the spatiotemporal expression patterns of *adar2* and *gria2α*, a homologue of *gria2* and a putative substrate of Adar2, were not identical. By quantitative RT-PCR analysis, the expression of *gria2α* has been reported to significantly increase after 30 hpf [16], while that of *adar2* mildly decrease (Fig. S1). Robust expression of *adar2* in the retina and cranial ganglia, especially the posterior lateral line ganglion/placode, started at 24 hpf (Fig. 1G), earlier than an overt expression of *gria2α* in these regions [18]. After 36 hpf, the *adar2* expression in the spinal cord and medulla oblongata diminished, while *gria2α* expression persisted (Fig. 1K and L). Moreover, *gria2α* expression has not been reported in the pharyngeal cartilages.

### Reduction of Q/R RNA editing of *gria2α* in *adar2MO*

**Figure 2:**
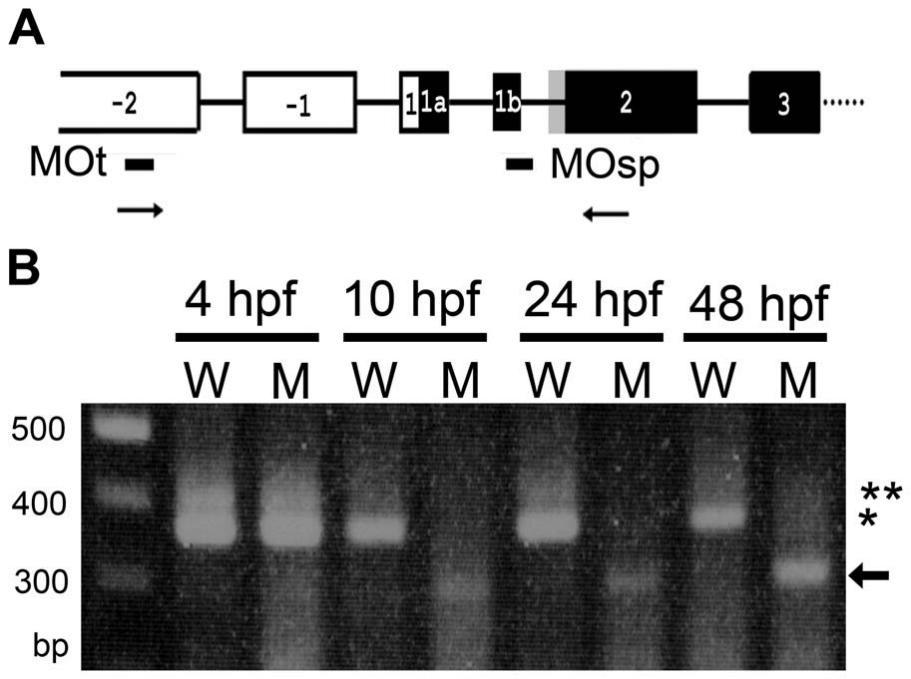
Injection of MOsp affects the splicing of *adar2* transcript. (**A**) 5′ gene structure of *adar2*. The gene structure is assigned by Slavov and Gardiner [14]. The non-translated and translated exons are respectively depicted in open and filled rectangular boxes. The gray box depicts the 47-bp alternatively spliced exon. Editing of the A upstream to the gray box results in the alternative splice to include the 47 bp sequence (+47 transcript). The locations of annealing sites of antisense morpholinos, MOsp and MOt, and PCR primers to check the effects of MOsp are indicated as lines and arrows below the gene structure, respectively. (**B**) Splicing of *adar2* is perturbed by injecting MOsp. RT-PCR was performed with RNA extracted from wild type (W) and *adar2MOsp* (M) of various development stages (hpf) shown on top of ethidium-bromide-stained agarose gel. An equal amount of cDNA that gives rise to the same amount of *actb1* amplicon was used to amplify *adar2*. Sizes of molecular markers are shown on the left side of the gel. Asterisk and double-asterisk respectively indicate the products of normal and edited (+47) *adar2*. Arrow indicates major aberrant splice products.

To reduce the expression of Adar2, two morpholinos, the MOt and MOsp, respectively inhibited translation and interfered the splicing of intron 1b, were injected into 1-cell zygotes (Fig. 2A), and the resultant morphants were respectively designated as *adar2MOt* and *adar2MOsp*. The efficacy of MOsp to perturb the splicing of *adar2* was estimated by RT-PCR (Fig. 2B). The splicing of *adar2* was not affected before midblastrula transition (4 hpf) when the *adar2* was maternally inherited. The maternal transcripts also included a slightly larger transcript which, as confirmed by sequence analysis, was the edited transcript that included the extra 47 nucleotides of intron 1b [14]. At 10 hpf, the normal-sized *adar2* transcript disappeared, and aberrantly spliced variants appeared in the *adar2MOsp* (Fig. 2B). Sequence analysis revealed that the aberrant splicing products mainly resulted from skipping exon 1b and occasionally from uses of cryptic donor sites in the exons 1a and -1. The normal-sized transcript represented 5 to 10% of the total (normal and aberrant) *adar2* transcripts between 10 to 72 hpf in the *adar2MOsp*. Normal-sized transcript increased to 15 to 25% at 96 hpf (data not shown), showing a reduced efficiency of MOsp to block splicing during larval development. We also noticed a reduction of *adar2* transcript (relative to *actb1*), presumably by failure to amplify unspliced and/or degradation of aberrantly spliced *adar2* transcript, in the *adarMOsp* (Fig. 2B). Quantitative RT-PCR analysis, by amplifying the 3′ end of *adar2* mRNA, confirmed that the relative amount of *adar2* significantly decreased in the 24 hpf *adar2MOsp* and, unexpectedly, also in the *adarMOt* (*p<*0.01, Table 1). The expression level and splicing of *adar2* were not affected in the *adar2MOc*, embryos receiving control morpholino (MOc) with 5 nucleotide substitutions of MOsp (Table 1).

**Table 1:** Quantitative analysis of gene expression^a^ at 24 hpf.

Genotype Gene^b^	*adar2MOc* (n = 3)^c^	*adar2MOsp* (n = 4)	*adar2MOt* (n = 7)	*adar2MOt-p53^AUG^* (n = 3)	*gria2αQRMO* (n = 3)
*adar2*	0.98±0.1	0.42±0.24*	0.62±0.1*	2.83±0.6**	0.79±0.14
*gria1α*	0.9±0.15	0.52±0.41	0.81±0.4	1.15±0.2	0.81± 0.14
*gria2α*	0.86±0.09	0.45±0.37	0.66±0.58	1.53±1.07	0.84±0.14
*cycg1*	1.38±0.14	6.04±4.52	9.18±2.17*	4.79±1.02*	3.2±0.63*
*mdm2*	1.17±0.14	3.09±1.9	3.66±0.5*	2.64±2.0	12.27±2.46*
*Δ113p53*	1.34±0.39	22.84 ± 18.32	20.1±7.8*	14.76±28.66	156.78±23.51*
3′-*p53*	1.2±0.16	4.07±2.77	5.5±0.39*	not determined	not determined
5′-*p53*	1.16±0.16	1.21±0.7	1.23±0.36	1.2±0.89	1.02±0.38
*neuroD*	0.96±0.06	0.39±0.25*	0.63±0.21*	0.92±0.1**	0.81±0.15
*neurog1*	0.92±0.06	0.57±0.31	0.81±0.11	1.04±0.11**	0.75 ± 0.14
*sox9a*	1.15±0.22	1.19±0.26	1.15±0.16	not determined	0.89±0.06
*sox9b*	1.08±0.1	0.64±0.01*	0.41±0.17*	not determined	0.55±0.05*

a : a relative gene expression level was determined and then normalized to the expression level of β-actin (actb1). The values (mean ± standard deviation) are the relative gene expression levels of morphants compared to those of the wild type (un-injected) of the same batch.

* indicates significant difference (p<0.05) between hypo-Q/R-editing morphants and adar2 control morphant (adar2MOc)

** indicates significant difference between adar2MOt-p53^AUG^ and adar2MOt.

b : Accession numbers are listed in Table S1. neuroD and neurog1 (neurogenin 1) are proneuron genes; ccng1(cyclin G1), mdm2 and Δ113p53 (a short isoform of p53 transcribed from an internal promoter) are p53-responsible genes. 3′-p53 and 5′-p53 respectively represent transcripts encoding all the isoforms of p53 and long isoform of p53. sox9a and sox9b are expressed in the neural crest cells and other cell types.

c : n, number of independent injection

The Q/R site of zebrafish *gria2α*, the predominant *gria2* paralogue expressed during embryonic and early larval development, is partially edited between 4 to 16 hpf [16]. The fraction of edited *gria2α^R^* decreased mildly but significantly in the *adar2MO* between 12 to 48 hpf, whereas it was unaffected in the *adar2MOc* (Table 2). Since the correctly spliced *adar2* transcript was translatable in the *adar2MOsp*, it was not surprising to observe that the fraction of *gria2α^R^* in *adar2MOsp* was higher than that in *adar2MOt* at 12 hpf. Overexpression of Adar2, by injecting *adar2* cRNA into one-cell zygotes, significantly enhanced the fraction of *gria2α^R^* in the *adar2MOt* and in the wild type (un-injected) embryos at 12 hpf, while overexpressing a mutant Adar2^cd^, with amino-acid substitutions in the catalytic domain, did not (Table 2). These results demonstrated that zebrafish Adar2, like mammalian Adar2, was capable of editing the Q/R site of *gria2α* and editing of the Q/R site was hampered in the *adar2MO*. At 24 hpf, the fraction of *gria2α^R^* in the *adar2MOt* and *adar2MOsp*, collectively referred to as *adar2MO*, was not overtly altered by the injection of *adar2* cRNA (Table 2). A cRNA encoding a catalytically active Adar2-GFP fusion protein was injected into one-cell zygotes to follow the expression of exogenous Adar2 during embryogenesis. The green florescence diminished before 20 hpf in 90% of the *adar2-GFP* RNA-injected embryos (data not shown), showing that the expression of exogenous Adar2 was transient. A short and transient expression of exogenous Adar2 explained why Q/R editing activity was not enhanced by Adar2 overexpression at 24 hpf.

**Table 2:** Efficiencies of Q/R RNA editing of gria2α.

Genotypes^a^	Developmental stages (hpf)	Normalized *gria2α^R^* frequency^b^ (sample number)
Wild type + Adar2	12	128.57% (2)
*adar2MOc*	12	103.93±2.03% (3)
*adar2MOsp*	12	91.72±4.31%* (5)
*adar2MOt*	12	75.82±18.33%* (5)
*adar2MOt* + Adar2	12	135.86±25.07%** (4)
*adar2MOt* + Adar2^cd^	12	55.05±25.07%* (4)
*gria2αQRMO*	12	37.25±32.2%* (3)
*gria2αQRMO* + Adar2	12	25.04% (2)
*adar2MOc*	24	102.94±2.55% (3)
*adar2MOsp*	24	69.81±18.74%* (4)
*adar2MOsp* + Adar2	24	82. 2% (1)^c^
*adar2MOt*	24	68.93±18.86%* (8)
*Adar2MOt* + Adar2	24	66.09% (1)^d^
*adar2MOt*-*p53* ^AUG^	24	75.71±11.48%* (3)
*gria2αQRMO*	24	18.59±11.43%* (6)
*gria2αQRMO*-*p53* ^AUG^	24	30.49±8.77%* (3)
*adar2MOt*	48	71.62 ±17.13% (5)
*gria2αQRMO*	48	25.02±6.31% (3)

a : morphants and protein expressed from the injected cRNA.

b : The fractions of edited *gria2α^R^* in the total *gria2α* transcript were determined and then normalized to that of the un-injected (wild type) embryos of the same batch. The fractions of *gria2α^R^* present in the 12-hpf wild type embryos varied from 50% to 65%, while it was between 96 to 98% in the 24-hpf and 48-hpf wild type embryos [18]. Values are represented as means ± standard deviation (number of independent injection). Statistics was analyzed by Student's *t*-test. Asterisk (*) indicates a significant difference (*p*<0.05) versus *adar2MOc*. Double asterisks indicate a significant difference versus *adar2MOt*.

C : the *gria2α^R^* frequency of the corresponding *adar2MOsp* was 81%.

d : the *gria2α^R^* frequency of the corresponding *adar2MOt* was 72%.

The R/G editing, controlling the recovery rate of AMPA receptors from desensitization, of *gria2* in the postnatal mice is also preferentially catalyzed by Adar2 [8]. The extents of R/G editing of *gria2α* were low (less than 15%) during zebrafish embryogenesis and early larval development. Overexpressing Adar2 could not enhance the editing of R/G site of *gria2α* in the 12-hpf wild type and *adar2MOt* embryos (data not shown). These results implied that either the Adar2 could not edit the R/G site of *gria2α* or the amount of Adar2 was not the key determinant of R/G editing activity during zebrafish embryogenesis as have been suggested in the developing mouse brain [19].

**Figure 3:**
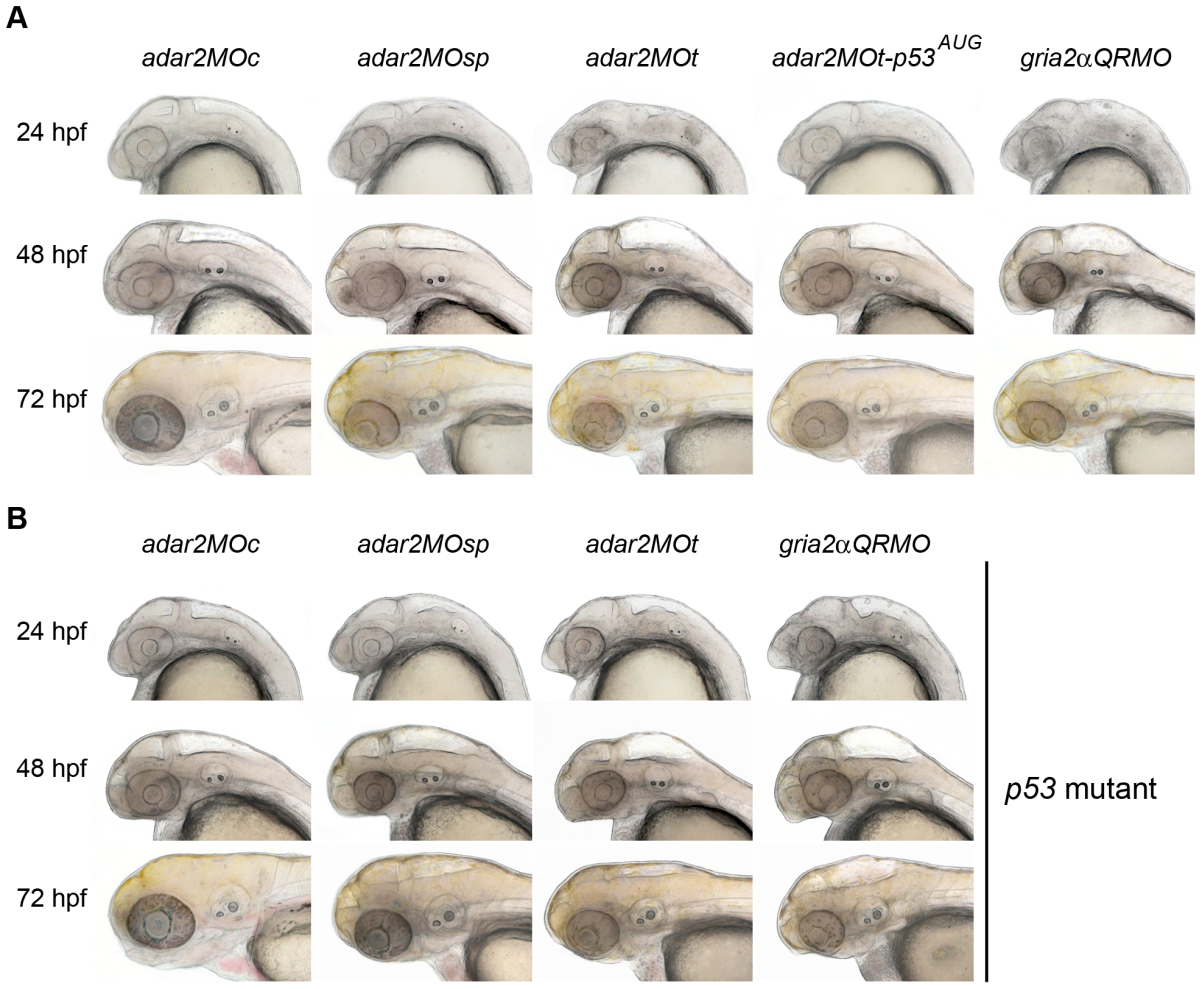
Head morphology of hypo-Q/R-editing morphants. (**A)** Bright-field images of morphants established in the wild type background between 24 to 72 hpf. The genotypes of the morphants are indicated on the top, where *adar2MOc, adar2MOsp*, *adar2MOt*, *adar2MOt-p53^AUG^* and *gria2αQRMO* respectively indicate embryos receiving the mismatch morpholino (MOc), splicing blocker (MOsp), translation blocker (MOt), MOt plus p53-MO^AUG^ [22], and RNA editing blocker (QRMO) morpholinos. Developmental time is shown on the left. The brain ventricles are enlarged and the size of heads is reduced in the hypo-Q/R-editing morphants. (**B**) Bright-field images of *p53* mutant (*tp53^zdf1^*) receiving morpholinos described in (A) between 24 to 72 hpf. The morphological changes of *adar2MO* and *gria2αQRMO* cannot be suppressed by losing p53 activity.

Despite a slight delay in development, the gross morphology of *adar2MO* appeared to be normal before 24 hpf (Fig. 3A). However, brain ventricles of more than 95% *adar2MO* became enlarged before 36 hpf (Figs. 3 and S2). The swollen diencephalic (DiV) and rhombencephalic (RhV) ventricles of 36-hpf *adar2MOt* were further confirmed by injecting rhodamine-conjugated dextran into the mesencephalic duct (Fig. S2). Hatching was 2- to 6-hour delayed in the *adar2MO*, and only 23 to 40% of the hatched *adar2MO* (4 batches, more than 35 embryos per batch) could completely escape from the chorion at 72 hpf. Larvae of *adar2MO* displayed tactile irresponsiveness and severe growth retardation after hatching.

### Specific blockage of the Q/R editing of *gria2α* in *gria2αQRMO*

Genetic studies have demonstrated that the failure to edit the Q/R site of *gria2* is responsible for the neuronal disorders and postnatal death observed in the *adar2* ^-/-^ mouse [8], [20]. To test if similar scenario existed between zebrafish *gria2α* and *adar2*, *gria2αQRMO* was generated by injecting the QRMO which paired to the exon complementary sequence (ECS) of *gria2α*. The fraction of edited *gria2α^R^* decreased drastically in the *gria2αQRMO*, showing that QRMO could efficiently block the Q/R editing of *gria2α* (Table 2). The fraction of *gria2α^R^* was unaffected by overexpressing Adar2 in the *gria2αQRMO* (Table 2), supporting that the effect of QRMO on blocking RNA editing was, as designed, *cis-*acting to disrupt the secondary RNA structure recognized by Adar2. Consistent to the previous observations of inefficient splicing of unedited *gria2^Q^* pre-mRNA in the editing-deficient mice [8], [10], the level of *gria2α* mRNA showed a trend of reduction, though not significantly, in the *adar2MO* (Table 1). Interestingly, the level of *gria2α* mRNA was not affected in the *gria2αQRMO* (Table 1), suggesting that the pairing of QRMO to intronic ECS might relieve the splicing hindrance by disruption the secondary RNA structure.

### Increased p53-dependent apoptosis in the hypo-Q/R-editing morphants

Similar gross morphological changes and locomotion defects were observed in the *gria2αQRMO* and *adar2MO*, collectively referred as hypo-Q/R-editing morphants (Fig. 3A and data not shown). Less than 20% of these morphants possessed a normal-sized pair of pectoral fins at 96 hpf, and morphant larvae developed edema in the pericardium and peritoneum cavities (data not shown). The hypo-Q/R-editing morphants died before 8 dpf (day postfertilization) with severe edema. These abnormalities were rarely observed in the control larvae of *adar2MOc* and un-injected wild type. Opaque areas, an indication of excessive cell death, were noticed in the head of hypo-Q/R-editing morphants (Fig. 3A). A survey of gene expression by differential display revealed an elevated expression of *cyclin G1* (*ccng1*), a p53-target gene, in the 24-hpf *adar2MOt*. Quantitative RT-PCR analysis confirmed that the expression levels of *ccng1*, *mdm2* and *p53-Δ113*, a truncated *p53* transcript initiated from an internal p53-dependent promoter [21], increased significantly in the 24-hpf hypo-Q/R-editing morphants but not in the *adar2MOc* (Table 1). The expression of full length *p53* transcript (5′-*p53*) was not significantly affected, indicating that only the *p53-Δ113* was up-regulated in the hypo-Q/R-editing morphants. Similar up-regulations of p53-responsible genes and elevated apoptosis have frequently been reported as the responses to stress and losses of cell-essential gene functions in zebrafish [21]–[23].

**Figure 4:**
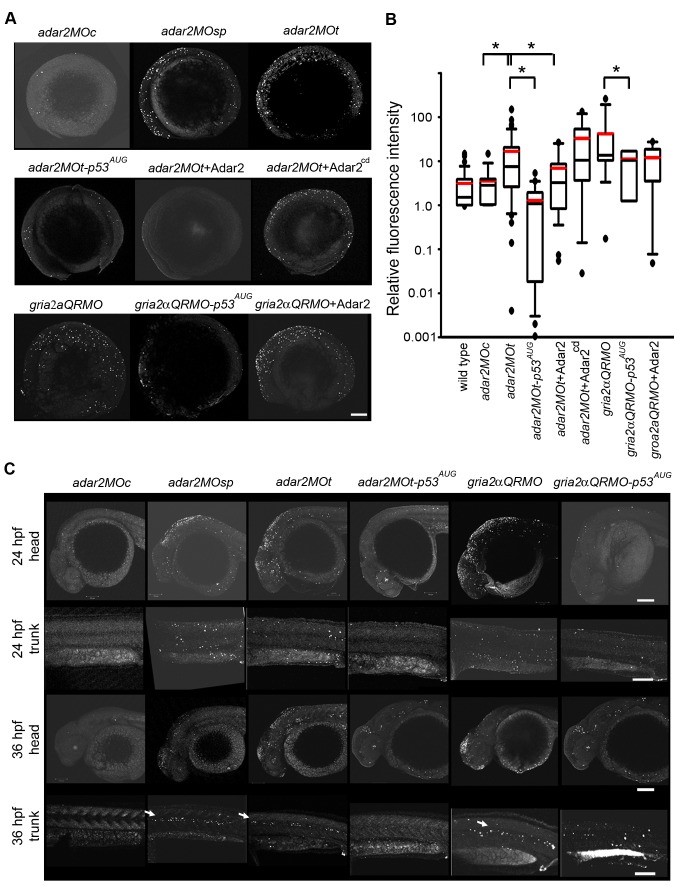
Increased p53-dependent apoptosis in the hypo-Q/R-editing morphants. (**A**) Apoptosis at the 5-somite stage. Representative images, except those co-injected with p53-MO^AUG^, were selected from the ones showing the mean fluorescence intensities in the TUNEL (terminal deoxynucleotidyl transferase-mediated dUTP nick end-labeling) analysis. The anterior is to the left, and dorsal side is to the top. Top panel shows the *adar2MOc*, *adar2MOsp* and *adar2MOt*. Middle panel shows *adar2MOt* co-injected with p53^AUG^ morpholino to block p53 activity, with *adar2* cRNA (Adar2) and with *adar2^cd^* cRNA (Adar2^cd^). The bottom panel shows *gria2αQRMO*, and the *gria2αQRMO* co-injected with p53^AUG^ and with *adar2* cRNA. (**B**) Tukey box plot of the relative TUNEL signals at 5-somite stage. Boxes represent 50% inter quartile values. Black and red lines respectively mark the median and mean intensities. Outliers are marked as dots. All signals were normalized to a wild-type embryo, showing mean intensity, stained in the same batch (relative fluorescence intensity). The total embryos included in the analysis are 8 *adar2MOc* (2 batches), 42 *adar2MOt* (9 batches), 19 *adar2MOt-p53^ AUG^* (3 batches), 20 *adar2MOt+*Adar2 (3 batches), 12 *adar2MOt+*Adar2*^cd^* (3 batches), 9 *gria2αQRMO* (3 batches), 7 *gria2αQRMO-p53^AUG^* (3 batches) and 5 *gria2αQRMO+* Adar2 (2 batches). (**C**) Increased p53-dependent apoptosis in specific regions of the 24- and 36-hpf hypo-Q/R-editing morphants. Lateral views of head and trunk at 24 and 36 hpf. Arrows indicate the apoptotic cells along the horizontal myoseptum. Scale bars represent 100 µm.

TUNEL (terminal deoxynucleotidyl transferase dUTP nick end labeling) assay was employed to quantify apoptosis in the morphants (Fig. 4). The epiblast of 5-somite-stage *adar2MO* and *gria2αQRMO* started to display a significantly more TUNEL signals than that of *adar2MOc* (Figs. 4A and B). At 24- and 36-hpf, apoptosis prominently occurred in the *adar2-* and *gria2α-*expressing regions, including eye, midbrain and hindbrain (Fig. 4C). In addition, apoptosis frequently happened along the horizontal myoseptum of 36-hpf hypo-Q/R-editing morphants (Fig. 4C). On the other hand, TUNEL-positive signals were rarely observed in the forebrain and spinal cord, where also expressed *adar2* and *gria2α* (Fig. 4C).

Since p53 activity was enhanced in the hypo-Q/R-editing morphants, we investigated if the apoptosis depended on p53 activity by co-injection of *p53* antisense morpholino. Co-injection of *p53-*MO^AUG^ (*adar2MOt-p53^AUG^* and *gria2αQRMO-p53^AUG^*), which blocks the translation of full-length p53 [21], [22], significantly suppressed the apoptosis in the hypo-Q/R-editing morphants (Fig. 4) without enhancing the fraction of *gria2α^R^* (Table 2). Results of Acridine orange staining also confirmed that a majority of the apoptosis depended on p53 activity (Fig. S2). However, the locomotion defects and morphological changes of the hypo-Q/R-editing morphants could not be reverted by co-injection of *p53-*MO^AUG^ (Fig. 3A) or morphants established in the *p53* mutant background (Fig. 3B). These results indicated that excessive cell death was not a major cause for the morphological and behavioral changes in the hypo-Q/R-editing morphants.

Off-target effect manifested as p53-dependent apoptosis, especially in the brain and spinal cord, is a major concern in studying gene function by morpholino knockdown technology [24]. Unlike cell death resulted from off-target effect of antisense morpholinos, apoptosis was rare in the forebrain and spinal cord of 24-hpf and 36-hpf *adar2MO* (Fig. 4C). RNA rescue experiments were performed to demonstrate that apoptosis was an on-target effect. Injection of *adar2* mRNA resulted in a significant suppression (*p* = 0.0007) of apoptosis in the epiblast of 5-somite *adar2MOt* (Fig. 4B). On the other hand, apoptosis was not suppressed by overexpressing the catalytically dead Adar2^cd^, indicating that the RNA editing activity of Adar2 was required for suppressing apoptosis in the *adar2MOt*. Moreover, overexpression of Adar2 did not enhance the *gria2α^R^* level in the *gria2αQRMO* and could not suppress apoptosis (Fig. 4B). There was an inverse correlation between the *gria2α^R^* levels at 12 hpf and the TUNEL signals on 5-somite stage among *adar2MO*, *adar2MO* overexpressing Adar2/adar2^cd^ and *gria2αQRMO* (Fig. 4B and Table 2).

### Abnormal development of the nervous system in the hypo-Q/R-editing morphants

**Figure 5:**
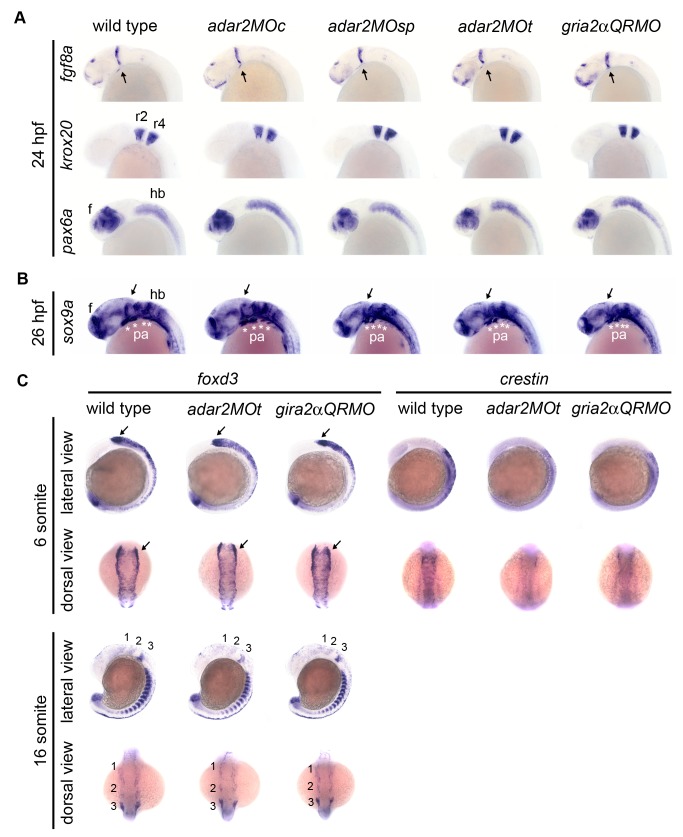
Gene expression in neural tube and migratory neural crest. **(A, B)** Embryos are under lateral view. **(A)** Expressions of brain regionalization genes. Expression of *fgf8*, *krox20*, and *pax6a* appear normal in the 24 hpf hypo-Q/R-editing morphants. (**B, C**) Expressions of neural crest genes. The expression of mesenchymal condensations marker, *sox9a*, in the pharyngeal arch (pa, *) are slightly but consistently reduced in the hypo-Q/R-editing morphants. Expressions of neural crest markers *foxd3* and *crestin* are mildly affected at 6-somite and 16-somite stages. Anterior is respectively to the left and top at lateral and dorsal views. 1, 2 and 3 are the three migration cranial neural crest streams originated from posterior mesencephalon and hindbrain. Arrows indicate the midbrain hindbrain boundary. e, eye; hb, f, forebrain, hb, hindbrain; r2 and r4, rhombomeres 2 and 4.

**Figure 6:**
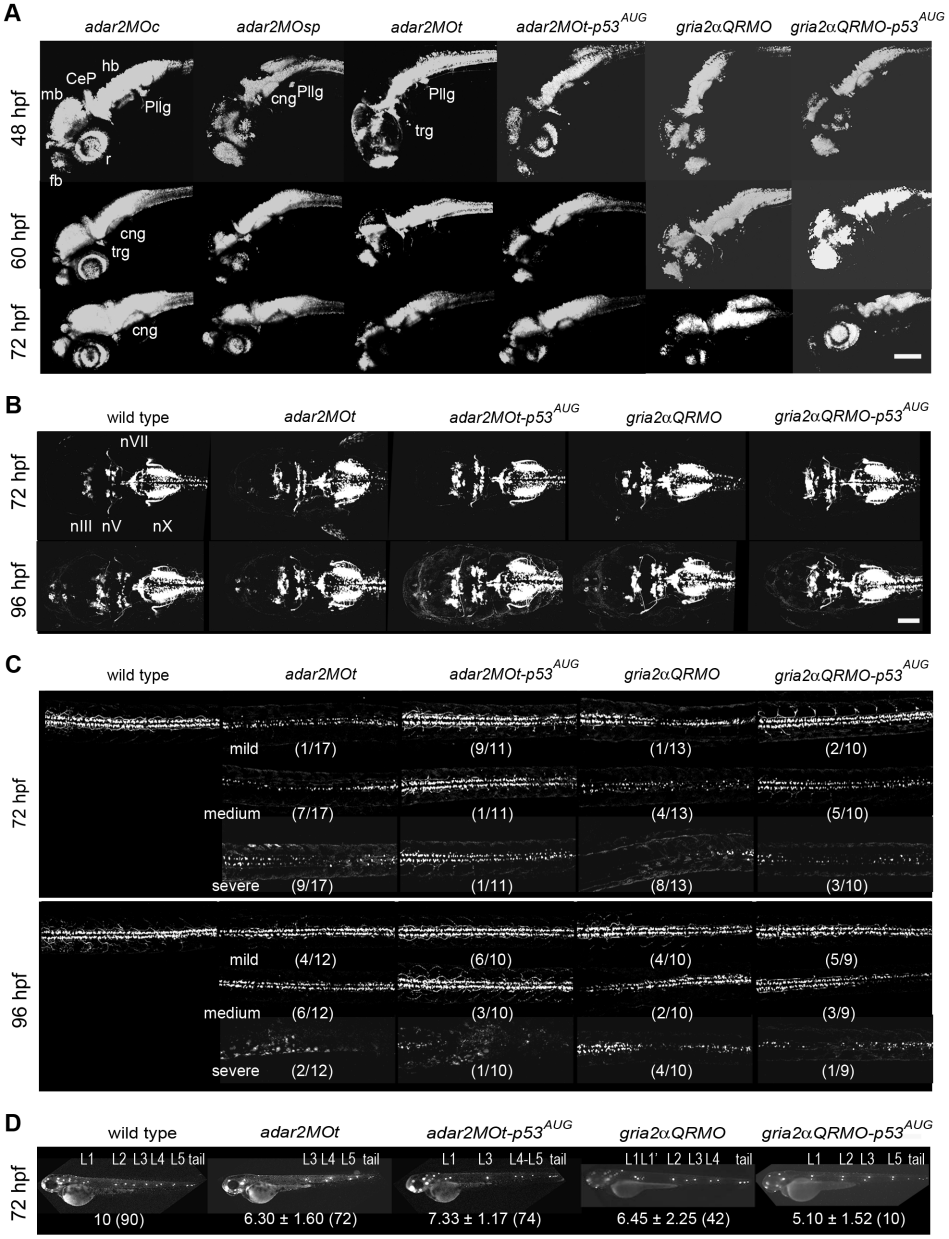
Defective development of the nervous system in the hypo-Q/R-editing morphants. (**A**) The development of early and mature neurons. Confocal microscopic observations of the kaede fluorescence in Tg(*HuC:kaede*) receiving morpholino injections. The kaede-expressing domain is reduced in the brain of hypo-Q/R-editing morphants. CeP, cerebellar plate; cng, cranial ganglion; fb, forebrain; hb, hindbrain; pllg, posterior lateral line ganglion; r, retina; trg, trigeminal neuron. (**B**) The development of cranial motor neurons. Confocal microscopic observations of the GFP in the heads of Tg(*isl1:GFP*) receiving morpholino injections. The cranial motor neurons are only mildly affected. nIII; oculomotor nuclei; nV; trigeminal nuclei; nX, vagus nuclei. (**C**) The development of spinal motor neurons. Confocal microscopic observations of the GFP in the trunks of Tg(*isl1:GFP*) receiving morpholino injections. The spinal motor neurons dorsal to the yolk extension are shown. The effects of morpholino treatments were classified into three groups by relative density of motor neuron in each treatment. The numbers in parenthesis indicate the numbers of larvae in a class over all the observed larvae. Scale bar represents 100 µm. (**D**) The development of lateral line neuromasts. Epifluorescent microscopic observations of the lateral line neuromasts stained by Di-4-Asp [48]. L1-L5 are the posterior lateral line neuromasts, and tail represents the tail neuromasts. L1′ is the secondary PLL neuromast. The average ± s.d. (number of larva) of the primary lateral line neuromasts are shown below. Larvae without tail neuromast were excluded from the analysis.

Since *adar2* and *gria2α*were highly expressed in the nervous system and apoptosis was elevated in selective brain regions of the hypo-Q/R-editing morphants, we investigated if neuronal development was affected. The *fgf8-*, *krox20*- and *pax6a*-expressing domains in the 24-hpf morphants were similar to that of the wild type embryos, indicating that neurulation and regionalization were not grossly affected in the hypo-Q/R-editing morphants (Fig. 5A). In addition, the dorsal expression of *sox9a* in the head of 26 hpf hypo-Q/R-editing morphants also seemed to be comparable to that of wild type (Fig. 5B). The expression level of proneuron gene, *neurog1*, was slightly reduced in the hypo-Q/R-editing morphants. The expression of *neuroD*, expressed in the neuronal precursor and neuroblast, was significantly (*p*< 0.05) reduced in the 24-hpf *adar2MO*; however, *neuroD* expression was only mildly affected in the *gria2αQRMO* (Table 1). We then examined the distribution of early and mature neurons by injecting morpholinos into Tg(*HuC:kaede*) line in which the *kaede* expression is driven by HuC promoter [25]. Coinciding to the regions with elevated apoptosis, the neuronal populations (kaede-expressing cells) of the retina, midbrain, and hindbrain of hypo-Q/R-editing morphants were consistently and severely reduced between 48 to 72 hpf, while that of the forebrain (fb) only became noticeably affected after 60 hpf (Fig. 6A). Although *adar2* was expressed in the Rohon-Beard neurons and interneurons of spinal cord, these neurons were not visibly affected in the hypo-Q/R-editing morphants before 48 hpf (data not shown). The reduction of neuronal populations in the brain was not reverted by a simultaneously reduction of p53 activity (Fig. 6A), showing that the reduction of neuronal populations was not resulted from excessive apoptosis in the hypo-Q/R-editing morphants.

Cell specific knock out mouse abolished *adar2* expression in subsets of motor neurons (the AR strain) displays delayed death of spinal cord motor neurons and selective cranial motor nerve nuclei, including trigeminal (nV) and hypoglossal (nXII) nuclei [20]. We investigated if the development of motor neurons was affected by injecting morpholinos into Tg(*isl1:GFP*) line where GFP is expressed in subsets of *isl1*-expressing neurons, namely the cranial motor nuclei, some sensory neurons and secondary spinal motor neurons [26]. At gross level, the cranial motor nuclei (nIII, nV, nVII and nX) were only mildly affected (Fig. 6B), while the spinal motor neurons were noticeably affected in the hypo-Q/R-editing morphants at 72 and 96 hpf (Fig. 6C). The spinal motor neurons of the hypo-Q/R-editing morphants were more severely affected at 72 hpf than that at 96 hpf. A decreased effectiveness of MOt and QRMO may be responsible for the replenishment of spinal motor neurons at 96 hpf. Interestingly, *p53* knockdown could consistently, though partially, suppressed the loss of spinal motor neurons (Fig. 6C). Co-injection of *p53-*MO^AUG^ increased the density of spinal motor neuron in the hypo-Q/R-editing morphants. All the *adar2MOt* lost more than 50% of their spinal motor neurons, while none of the *adar2MOt-p53^AUG^* did. Similarly, the occurrence rate of losing more than half of motor neurons was reduced from 92% (12 out of 13) in the *gria2αQRMO* to 30% (3 out of 10) in the *gria2αQRMO-p53^AUG^*.

Both *adar2* and *gria2α* are expressed in the cranial ganglion (cng) and posterior lateral line ganglion/placode (pllg, Fig. 1 and [18]). The cng and pllg domains in the Tg(*HuC:kaede*) line was consistently reduced in the hypo-Q/R-editing morphants (Fig. 6A). The development of primary lateral line neuromasts, whose axons connected to the pllg, was studied. The migration of neuromast primodium was slightly delayed but not completely arrested in the hypo-Q/R-editing morphants. At 72 hpf, more than 95% of the hypo-Q/R-editing morphants possessed tail neuromasts. Wild-type (un-injected) and *adar2MOc* respectively possessed averages of 10 (n = 90) and 9.99±0.57 (average ± S.D., n = 90) primary PLL neuromasts at 72 hpf (L1-L5 of Fig. 6D). The number of PLL neuromasts on the hypo-Q/R-editing morphants decreased significantly and only less than 10% of the 72-hpf morphants developed 9 or 10 primary neuromasts. The first pair (L1) of PLL neuromasts usually appeared on the 5^th^ and 6^th^ myotomes of wild-type and *adar2MOc*, whereas that of the hypo-Q/R-editing morphants appeared on a broader region, from the 5^th^ to the 8^th^ myotomes or even on more posterior myotomes (Fig. 6D). The spacing between the L1 and L2 neuromasts was wider in the hypo-Q/R-editing morphants. These results indicated that the periodic deposition and perhaps differentiation of PLL neuromasts were perturbed in the hypo-Q/R-editing morphants. Despite a delay, secondary PLL neuromasts appeared in older morphants. The number of anterior lateral line (ALL) neuromasts was also reduced in the hypo-Q/R-editing morphants. Simultaneously knocking down the *p53* could not re-establish the periodic depositions of PLL and ALL neuromasts in the hypo-Q/R-editing morphants (Fig. 6D).

### Malformation of the cranial cartilages in the hypo-Q/R-editing morphants

**Figure 7:**
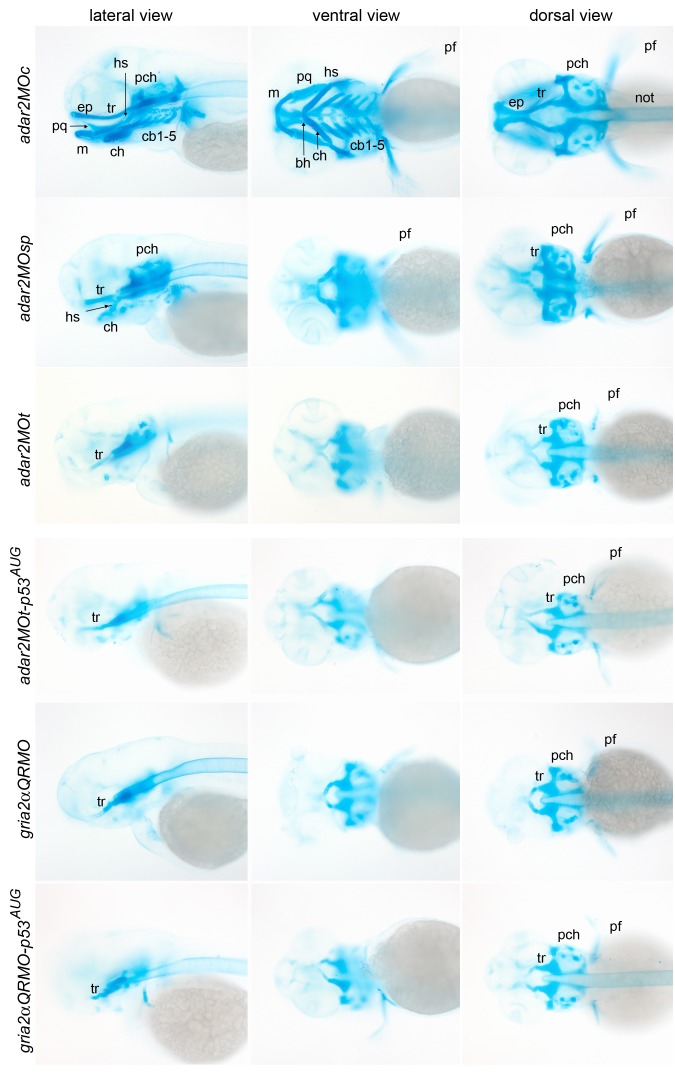
Malformation of the cranial cartilages of hypo-Q/R-editing morphants. Representative images of Alcian blue staining of the head cartilages are presented in three views. Ventral view is taken at a deeper focus from the dorsal side. Abbreviations: bh, basihyal; cb, ceratobranchials; ch, ceratohyal; ep, ethmoid plate; hs, hyosymplectic; m, Meckel's cartilage; not, notochord; pch, parachordal; pq, palatoquadrate; tr, trabeculae.

Since a high level of *adar2* was expressed in the posterior pharyngeal arches (ceratobranchials, cb1-5, Fig. 1O and P’), head cartilages of the *adar2MO* were stained by Alcian blue. The cartilaginous head skeletons were severely malformed in the *adar2MO* but not in the *adar2MOc* (Fig. 7). The pharyngeal arches have not been reported to express *gria2α*. Unexpectedly, the head cartilages of *gria2αQRMO* displayed similar defects as those of *adar2MO*. In general, pharyngeal skeletons (ventral view) were more severely affected than the neurocranium (dorsal view), and the anterior neurocranium was more severely affected than the posterior one (Fig. 7). The pharyngeal skeletons completely disappeared from the *adar2MOt* and *gria2αQRMO*, while rudiments of posterior pharyngeal skeletons, including ceratohyal (ch) and posterior pharyngeal arches (cb1-5, 3^rd^ to 7^th^ arches), remained in the *adar2MOsp*. In the hypo-Q/R-editing morphants, the anterior ethmoid plate (ep) was completely deleted but the trabeculae (tr), parachordal (pch) and pectoral fins were merely shortened and reduced. The defective development of cranial cartilages was not reverted by reducing the p53 activity.

### Neural crest defects in the hypo-Q/R-editing morphants

Cranial cartilages are derived from the neural crest of head [27]. Genes expressed in the neural crest cells were studied by WISH. Though the dorsal expression of *sox9a*, a marker of cranial mesenchymal condensations, seemed to be normal, the ventral *sox9a* expression in the pharyngeal arch (pa) was mildly but consistently reduced in the 26-hpf hypo-Q/R-editing morphants (Fig. 5B). Pharyngeal arches are colonized from three *foxd3-* and *crestin-*expressing streams of migration cranial neural crest cells originated from the posterior midbrain and hindbrain [27]. The expression of neural crest specifier, *foxd3*, was not affected in the 6-somite morphants (Fig. 5C). Nevertheless, we noticed the neural plate boarders of the hypo-Q/R-morphants were narrower than that of the control embryos. The expression of *crestin* in the early migration neural crest cells was mildly reduced at the 6-somite stage (Fig. 5C). These results suggested that the pre-migratory neural crest cells were not severely affected in the hypo-Q/R-editing morphants.

**Figure 8:**
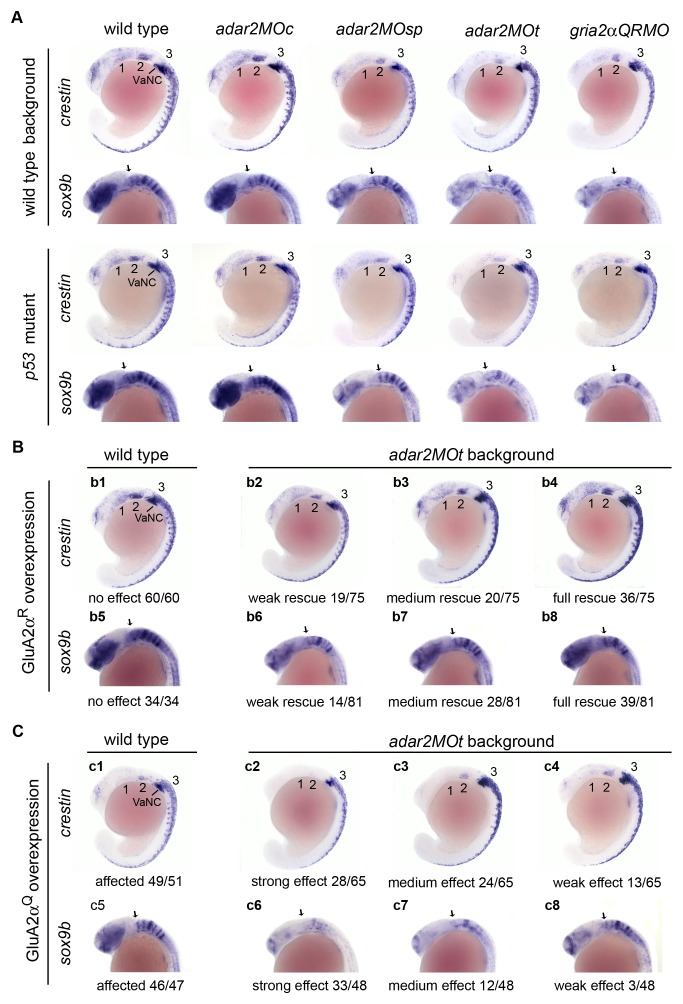
Increased GluA2α^Q^ is sufficient to induce the neural crest defects observed in the adar2MOt. Lateral views of 18-somite stage embryos. (**A**) Expressions of crestin and sox9b in the hypo-Q/R-editing morphants are affected. Morphants created in the wild type and p53 mutant show negligible differences, indicating that the expression defects in the hypo-Q/R-editing morphants are p53-independent. (**B**) Overexpression of GluA2α^R^ partially restores the expressions of crestin and sox9b in the adar2MOt. Injection of gria2α^R^ cRNA into the wild type zygotes does not alter the expressions of crestin and sox9b (b1 and b5). However, injection of gria2α^R^ cRNA into the adar2MOt fully or partially rescues the expressions. Effects of rescue range from weak (b2 and b6), medium (b3 and b7) to full (b4 and b8). Weak rescue of crestin expression is defined by slightly enhanced expression in the first (1) and second (2) streams of migration neural crest cells (b2), medium rescue is defined by enhanced anterior expression (b3), and full rescue is defined by restoring the wild type expression pattern and level. Rescue of sox9b expression is classified by the enhancement of overall sox9b expression (b6-b8). Rescue efficiencies are indicated. (**C**) Overexpression of GluA2α^Q^ affects the expressions of crestin and sox9b. The crestin and sox9b expressions in gria2α^Q^ cRNA-injected embryos (c1 and c5) are affected as that of adar2MOt. The crestin and sox9b expressions are further reduced in adar2MOt by overexpressing GluA2α^Q^. The additive effect varies from strong (c2 and c6), medium (c3 and c7), to weak (c4 and c8) further reduction of crestin and sox9b expressions. The occurrence rate of each phenotype is shown. 1, 2, 3, the first, second and third steams of migration cranial neural crest; e, eye; ep, epiphysis; f, forebrain; hb, hindbrain; VaNC, vagal neural crest.

Beginning at 16-somite stage, the *foxd3* expression in the 3 migration neural crest streams was reduced in the hypo-Q/R-editing morphants (Fig. 5B). At 18-somite stage, the *crestin* expression in the first and second streams was severely reduced in the hypo-Q/R-editing morphants, while *crestin* expression in the third stream, vagal neural crest and trunk neural crest was mildly affected (Fig. 8A). In addition, the *crestin*-expressing cells on the trunk of hypo-Q/R-editing morphants did not migrate as far ventrally as that of the wild type and *adar2MOc* embryos (Fig. 8A). The neural crest and cartilage defects of hypo-Q/R-editing morphants resemble to that of the *sox9b* mutant [28]. Not surprisingly, the *sox9b* expression in forebrain, epiphysis, eye and rhombomere boundaries in the hindbrain of the hypo-Q/R-editing morphants were reduced (Fig. 8A). The overall reduction of *sox9b* expression in the hypo-Q/R-editing morphants was confirmed by quantitative PCR analysis (Table 1). Since the segmentation of rhombomeres seemed to be normal, as evident of the expression pattern of *krox20* (Fig. 5A) and of morphological observations (data not shown), the reduction of *sox9b* expression in the rhombomere boundaries could not be attributed to structural defect of hindbrain. Reduction of p53 activity could not restore the normal expressions of *crestin* and *sox9b* in the hypo-Q/R-editing morphants.

### Hypo-Q/R editing of *gria2α* is sufficient to render the neural crest defects

The defective expressions of *foxd3*, *crestin* and *sox9b* suggested that migration of neural crest cells were affected in the hypo-Q/R-editing morphants. The involvement of Q/R editing of *gria2α*in the neural crest development was further studied. Overexpressing the edited GluA2α^R^, by injecting *gria2α^R^* cRNA into one-cell zygote, exerted no advert effect on the *crestin* and *sox9b* expressions in the wild type (Fig. 8B, b1 and b5). Overexpressing GluA2α^R^ in the *adar2MOt* partially or completely restores (∼48%) the expression patterns of *crestin* and *sox9b* (Fig. 8B, b2-b4 and b6-b8), showing that the neural crest defect of the *adar2MOt* could be rescued by supplementing *gria2α^R^*, the catalytic product of Adar2. Most importantly, overexpressing GluA2α^Q^ in the wild type background affected the expressions of *crestin* and *sox9b* (Fig. 8C, c1 and c5) in a similar manner as those observed the hypo-Q/R-editing morphants. Moreover, the effects of overexpressing GluA2α^Q^ was additive to the *adar2* knockdown (*adar2MOt*, Fig. 8C, c2-c4 and c6-c8). Compare to *adar2MOt*, the expressions of *crestin* and *sox9b* were respectively further reduced in 80% (52/65) and 93% (45/48) of the *adar2MOt* overexpressing GluA2α^Q^. This result supported that the neural crest cells defects observed in the hypo-Q/R-editing morphants resulted from an increased level of GluA2α^Q^.

## Discussion

### Adar2 edits the Q/R site of *gria2α*

Introducing antisense morpholinos to block the translation (MOt) and splicing (MOsp) of adar2 significantly reduces the editing of AMPA receptor (AMPAR) subunit gria2α at Q/R site, while overexpressing Adar2 enhances the editing of the same site (Table 2). These results support that zebrafish Adar2 edits the Q/R site of AMPAR subunit gria2α in vivo. Conversely, overexpressing zebrafish Adar1 cannot enhance Q/R editing of gria2α (Chou, unpublished result), suggesting that, in the 12- and 24-hpf wild type and adar2MOt embryos, the Q/R site is not efficiently edited by Adar1. In addition to Adar1, the second zebrafish Adar2 paralogue (Adar2b), which displays 89% of sequence similarity to the Adar2 [29], may complement the Q/R editing activity and account for the high levels of edited gria2α^R^ in the adar2MO (Table 2). Furthermore, the two morpholinos, MOt and MOsp, may not fully block the expression of adar2, and a residual Adar2 activity efficiently edits the Q/R site of gria2α.

Elevated GluA2α^Q^ is sufficient to render the neural crest defect and may be responsible for other defects observed in the adar2MO

Although there are zebrafish morphants/mutants exhibiting some of the developmental defects, namely reducing neuronal populations of head, deformed cartilages and the irregular deposition of PLL neuromasts, as those observed in the adar2MO, none of the morphants/mutants that we are aware of display all of these defects [23], [28], [30]. On the other hand, the gria2αQRMO, in which the Q/R editing of gria2α was specifically blocked by QRMO paired to the intronic ECS, displays almost an identical spectrum of morphological and developmental changes in the nervous system and cranial cartilages as that of adar2MO. These changes vary slightly in severity but involve identical regions of the brain, spinal cord and head cartilages at matching stages. The highly similar phenotypic changes of zebrafish adar2MO and gria2αQRMO are suggestive that the same function/pathway is perturbed in these animals. Reminiscing to that GluA2^Q^ is attributed to the similar neurological changes observed in the adar2^−/−^ and gria2^+/ΔECS^ mice [8], [10], it is highly plausible that an elevated level of GluA2α^Q^ accounts for a majority, if not all, of the defects in adar2MO. This possibility is supported by the observations that overexpressing the GluA2α^R^ can restore the expression patterns of crestin and sox9b in the adar2MOt (Fig. 8B) and overexpressing the GluA2α^Q^ induces wild type embryos to display neural crest defects as those observed in adar2MOt (Fig. 8C). These observations unequivocally demonstrate that an increase of GluA2α^Q^ level is sufficient to render the neural crest defect. Although that elevated GluA2α^Q^ is responsible for the impaired development of the nervous system in the adar2MO has not been fully proven in this study, this possibility is consistent to the reported GluA2^Q^ effects on neuronal death and neuronal activities [8], [10], [20], [31], [32]. Interestingly, the two types of hypo-Q/R-editing morphants, adar2MO and gria2αQRMO, display identical developmental defects while the levels of the unedited gria2α^Q^ greatly differ (Table 2). We speculate that the aforementioned defects are related to the enrichment of GluA2α^Q^ in tissues/cells expressing only adar2 but not adar2b.

A majority of the developmental defects observed in the hypo-R/Q-editing morphants is not attenuated by inhibiting apoptosis

In addition to the developmental defects, excessive p53-dependent cell death is consistently observed in regions, including the eyes, midbrain, hindbrain and horizontal myoseptum, in the hypo-Q/R-editing morphants (Fig. 4). Overexpressing the Adar2 suppresses apoptosis in the adar2MO, demonstrating that reducing the Adar2 activity leads to apoptosis in the adar2-expressing cells/tissues. Excessive apoptosis has also been observed in the adar1-expression tissues of adar1-deficient mouse [7], [12]. Unlike the phenotypic changes observed in zebrafish deficient of essential gene functions [23], suppressed apoptosis by p53-inactivation cannot replenish the neuronal populations of brain nor restore normal cranial cartilage development in the hypo-Q/R-editing morphants (Figs. 6–8). Therefore, excessive apoptosis alone could not fully account for the developmental abnormalities observed in the hypo-Q/R-editing mutants. Decoupling cell death and developmental defects is not uncommon. For example, the neural crest cell migration and cartilage development defects of arl6ip1 morphant are p53-independent and cannot be reverted by suppressing apoptosis [33]. There is a good correlation between brain regions showing excessive apoptosis and reduced neuronal populations, implying that apoptosis and impaired development are induced by a common mechanism in the hypo-Q/R-editing morphants. It remains to be determined whether the apoptosis in brain is triggered by the perturbed development or is independently induced.

Excitotoxicity has been proposed to be responsible for the loss and death of selective neurons in the mouse mutants deficient of Q/R editing of gria2 [8], [10], [20], [31], [32]. Although the involvement of p53 in the loss of neurons in the Q/R editing-deficient mice has not been examined, p53 is responsible for brain damage induced by seizure and for neuronal death by excitotoxicity [34], [35]. It remains to be determined if excitotoxicity is responsible for the apoptosis in neurons/progenitor cells and non-neuronal cells, for example cells along the horizontal myoseptum at 36 hpf, of the hypo-Q/R-editing morphants (Fig. 4C).

In the hypo-Q/R-editing morphants, knocking down p53 activity partially suppresses the loss of motor neurons (Fig. 6C). Slow loss of spinal motor neurons has been shown in the AR mouse losing Adar2 function in motor neurons [20]. Although the possibility of delayed loss of motor neurons cannot be excluded, there is no evidence to support that cell death occurs at isl1-expressing spinal motor neurons over a period of 2 days (72 to 120 hpf) in the hypo-Q/R-editing morphants. It is speculated that the reduction of motor neurons at 72 hpf may result from a p53-dependent reduction of progenitor cells in the hypo-Q/R-editing morphants.

### The effects of increasing unedited GluA2α^Q^ in the zebrafish development

Unlike the Q/R editing-deficient mice, the hypo-Q/R-editing morphants are not seizure-prone but display locomotion defects. In addition, the zebrafish hypo-Q/R-editing morphants display gross anatomical defects not present in the Q/R editing-deficient mice [8], [10], [36]. Why do the phenotypes of adar2^−/−^ mouse and zebrafish adar2MO differ while editing of the same substrate, namely the Q/R site of gria2/gria2α, is involved in both animals? One likely explanation is that zebrafish adar2 and gria2α have novel expression sites such as the adar2 expressed in the pharyngeal arches (Fig. 1O). Since migration of PLL neuromasts and neural crest cells are affected by hypo-Q/R editing, the unedited GluA2α^Q^ may exert novel activities on the maintenance, differentiation and/or migration of neurons and cranial neural crest in the zebrafish through increasing the activity of GluA2-containing Ca^2+^-permeable AMPARs [9], [31]. The GluA2^Q^-containing Ca^2+^-permeable AMPAR has been reported to direct the differentiation of cultured human neuronal progenitor cells [37]. Furthermore, the study of plant ionotropic glutamate receptor and the wide distribution of mammalian glutamate receptors outside the nervous system have raised the possibility that glutamate receptors may mediate cell to cell communication [38], [39]. The phenotype of the hypo-Q/R editing zebrafish is the first in vivo study to show that an increase of unedited GluA2α^Q^ grossly affects the migration of cranial neural crest cells. The possibility that GluA2-containing AMPARs mediate cell to cell communication, besides synaptic transmission, during zebrafish embryogenesis warrants further investigation.

### Functions of zebrafish Adar2

On the basis of the phenotypic changes observed in the adar2MO, the Adar2 activity is required for normal zebrafish development. Many of the developmental defects and excessive cell death in the adar2MO are most likely resulted from elevating the unedited gria2α^Q^ level. Mouse Adar2 is known to edit a plethora of RNA sequences including protein-coding and non-protein coding sequences, as well as miRNA [3], [6]. Not surprisingly, other phenotypic changes and an alternation in the transcriptome have been described in the adar2^−/−^/gria2^R/R^ mouse that carry chromosome-encoded gria2^R^ to suppress the neurological defects and lethality resulted from the adar2-deficient. These changes are not related to the Q/R editing of gria2 and are attributed as direct and indirect consequences of altering RNA editing activity [36]. Among the few gene expression reported here, the adar2 and neuroD mRNA levels in the adar2MO and gria2αQRMO are differentially affected (Table 1). Transcriptome changes in the adar2MO may arise from the consequences of elevated GluA2α^Q^, namely the developmental defects and cell death reported here, or functions unrelated to gria2α editing. Moreover, the similar but not identical spatiotemporal expression patterns of adar2 and gria2α (Figs. 1 and S1) suggest that cells affected by adar2 knockdown (adar2MO) and by reducing Q/R editing of gria2α (gria2αQRMO) may not be the same. Given the highly similar phenotypes between the adar2MO and gria2αQRMO, we speculate that cells/tissues of the same lineages, but may not be at the same differentiation stage, are affected in these hypo-Q/R-editing morphants during embryogenesis. Therefore, the development and/or survival of neuroD-expressing neuroblast cells may be more severely affected in the adar2MO than those in the gria2αQRMO, resulting in a significant decrease of neuroD expression in the adar2MO. Similarly, decrease adar2 level may also be resulted from excessive cell death in the adar2-expressing cells in the adar2MO (Table1). These possibilities are consistent with our observation that p53 inactivation enhances the adar2 and neuroD levels in the adar2MOt (Table 1). Degradation of the improperly spliced adar2 pre-mRNA further reduces the adar2 level in the adar2MOsp. The reduction of gria2α expression in the adar2MO may be a combined effect of inefficient splicing of unedited gria2α^Q^ pre-mRNA and abnormal differentiation/cell death of gria2α and adar2 co-expressing cells. The relatively high variability of the gria2α levels in the adar2MO, when compared with that of gria2αQRMO (Table 1), may reflect the defective differentiation/cell death of gria2α-expressing cells is influenced not only by the elevated GluA2α^Q^ but also by other, possibly more chaotic, factors in the adar2MO. Similarly, the levels of gira1α, encoding AMPAR subunit GluA1a and frequently co-expressed with gria2α [18], also show high variability in the adar2MO, but the levels of gria1α correlated well with gria2α in each independent treatments (Table 1). In the future, it will also be of interest to know if zebrafish Adar2 RNA editing activity may also contribute to neuronal protection as suggested by the study of adar2^−/−^/gria2^R/R^ mouse [36]. Consequently, results of this study does not exclude the possibility that Adar2 also catalyzes the editing of other sites/RNA, such as miRNAs, and in turn to modulate the development of zebrafish larvae [3], [40], [41].

## Materials and Methods

### Zebrafish strains and generation of morphants

Zebrafish (*Danio rerio*, Oregon AB line), p53 mutant (tp53^zdf1^) [42], Tg(*HuC:kaede*) and Tg(*islet1:GFP*) lines, kind gifts of Dr. H. Okamoto [25], [26], were provided by Taiwan Zebrafish Core Facility. Crosses of female Tg(*HuC:kaede*) and Tg(*islet1:GFP*) to male Oregon AB lines were respectively used to examine the populations of neurons and the motor neurons. Reciprocal crosses yielded similar results. One-cell zebrafish zygotes were collected 15-min after the beginning of the light cycle and defined as 0 hpf. Times of development were expressed as hour postfertilization (hpf) and day postfertilization (dpf) at 28.5°C. Morphological criteria, as described by Kimmel et al. [43], were used to select embryos at specific stages. Morphants of later stages (36–96 hpf) were also selected by morphology at 36 hpf. Only morphants with mildly swollen brain ventricles and straight body plane were used for later studies. In most experiments 0.003% N-phenylthiourea (Aldrich) was added at 12 hpf to inhibit pigment formation. Tricaine (3-aminobenzoic acid ethylester, Sigma) was used to anesthetized embryos and larvae when live imaging was applied. The permits for animal care and experiments were obtained from the Committee for Experimental Animals of National Tsing Hua University in agreement with the guidelines of Ministry of Agriculture of Taiwan.

Morpholino oligonucleotides were injected into one-cell embryos using an IM300 microinjector (Narishige Japan). The sequences (5′ to 3′) of antisense morpholino oligonucleotides (Gene Tools, LLC, Oregon) targeted against *adar2* were the translation blocker, MOt (GAAGACGTATGCGGTAAATGGCGAAA); the splicing blocker, MOsp (CAAGACAACAAAACACTCACTCAAG); and the 5-nt mismatched oligo, MOc (CAACACAACAATAGACTGACTGAAG). QRMO (TATGCAGCCGAAACACGGTACCACT) designed to complement the sequence of exon complementary sequence (ECS) within the intron downstream to the Q/R editing site of *gria2α* was used to block the Q/R editing of *gria2α*. Various doses of morpholino oligonucleotides were tested for their effects on producing consistent phenotypes. The final dose chosen for MOt was 6.4 ng per egg; while that for MOsp, MOc and QRMO were 8 ng per egg. At these doses, penetrance (enlarged ventricles) at 36 hpf was more than 95% and body deformation at 72 hpf was less than 1%. The p53-MO^AUG^ (GCGCCATTGCTTTGCAAGAATTCG) targeted against the translation initiation site was synthesized according to the published sequence [22]. The morphology of morphants was visualized after mounting embryos in low melting agarose. Bright-field microscopic images were taken with Nikon SMZ-U stereomicroscope or Zeiss Axioplan 2 equipped with Nikon Coolpix 990 camera. Images of the kaede and GFP-expressing domains in the Tg(*HuC: kaede*) and Tg(*islet1:GFP*) were taken by confocal microscope (LSM510, Zeiss) and complied.

### Cloning and Site-directed mutagenesis

Complementary DNA of *adar2*, containing a complete open reading frame and lacking the 5′-UTR sequence complementary to MOt, was amplified by RT-PCR and cloned to pBlueScriptII according to the sequence information (NM_131610). This clone was sequenced and served for further manipulations. The catalytically inactive mutant of Adar2 (Adar2^cd^) was created by site-directed mutagenesis (QuickChange, Statagene). The sequences (5′ to 3′) of the two primers were forward primer: ACGACTGCCATGCTGCATCATCGCACGGCGCTCA and reverse primer GCGCCGTGCGATGACTGCAGCATGGCAGTCGTTA to create the E^415A^I^416V^ mutation of the first active site of catalytic domain [44]. The Adar2-GFP fusion construct was created by first putting the entire *adar2* ORF, omitting the stop codon, into the *BamH*I site of peGFPC2. Then the Adar2-GFP coding region was cloned to T7TS. The *gria*2*α^R^* increased to 108% in the 12 hpf *adar2MOt* expressing Adar2-GFP. Complementary DNA of *gria2α^R^*, encoding the long C terminal isoform, was amplified by RT-PCR and cloned to pBlueScriptII according to the sequence information XM_005170898. Sequence analysis revealed that the clone was the flip isoform with edited R codon at the Q/R site and unedited R codon at the R/G editing site. The unedited *gria2α^Q^* was created by site-directed mutagenesis. The sequences (5′ to 3′) of the two primers were forward primer: AATATCGCATCCCTGCTGCATAAAAGCGCCCAG and reverse primer CTGGGCGCTTTTATGCAGCAGGGATGCGATATT. Complementary DNA was cloned to pT7TS. Capped cRNA was synthesized by *in vitro* transcription (mMachine, Ambion) as suggested by manufacturer. Each egg received 400 pg of cRNA.

### Whole-mount in situ hybridization

Whole-mount *in situ* hybridization (WISH) was performed as previously described [45]. Embryos were fixed in 4% phosphate-buffered paraformaldehyde (PFA/PBS, Merck). The embryos were rehydrated and treated with proteinase K for RNA probe penetration. The 1.5-kb sequence for synthesizing *adar2* riboprobe, comprising of the RNA binding domains, catalytic domain and 3′-UTR, was amplified by primers AACATGCAGCTGGACCAAACAC and AACAGAGACAAAAAAGGTGTGTGGAG, and cloned to pBlueScriptII. Antisense riboprobe was labeled with digoxigenin (Roche), recognized by alkaline phosphatase-conjugated anti-digoxigenin antibodies (Roche) and stained with NBT/BCIP (Roche). Zeiss AxioImager.M1 microscope and Zeiss AxioCam HRc camera were used to visualize and captured the images. Multiple images were combined with Adobe Photoshop CS2 software.

### Quantitative RT-PCR analysis and Q/R RNA editing assay

RNA was extracted from 0 hpf (40 eggs), 4 hpf (40 embryos), and 30 embryos of later developmental stages using RNeasy kit (Qiagen). One-third of the RNA was reverse transcribed by SuperScript III reverse transcriptase (Invitrogene) using oligo-d(T) and random hexamer as primers. The amount of *actb1* (β-actin) present in the cDNA was determine [16] and served as internal control for the efficiency of cDNA synthesis. An equal amount of cDNA, relative to the amount of *act1b*, was used to check for the efficiency of MOsp to block the splicing of *adar2* pre-mRNA. The primer sequences for checking the efficiency of blocking *adar2* splicing by MOsp were GCATAATTAAAGTCGGCTGTGATT and AGGCCGGAATTTGGAGTGTC, and the locations of the annealing sites are shown in Fig. 2A. The gene expressions at 24 hpf (Table 1) were determined by SYBR-Green real-time PCR (Applied Biosystems PRISM 7500) analysis and the results were fitted to previously established curves. Sequences of real-time PCR primers are listed in Table S1. The sequences of PCR primers were chosen from the 3′ ends of the transcripts and annealed to two adjacent exons. The amounts of transcripts were then normalized to the amount of *actb1* transcript. For a comparison among independent treatments, the normalized gene expression levels were expressed as relative gene expression levels by a second normalization to the respective gene expression levels of the wild type (Table 1).

The fraction of *gria2α^R^* was determined by a real-time PCR method [17]. Briefly, the *gria2α* cDNA was amplified by KOD-plus DNA polymerase and purified. The amounts of *gria2α^Q^* and total *gria2α* (*gria2α^Q^* plus *gria2α^R^*) were respectively determined by allele-specific and gene-specific primers. The fractions of *gria2α^R^* measured from the 24-hpf and 48-hpf wild type embryos were respectively around 96.5% and 98%, and that from the 12-hpf wild type embryos varied from 50% to 65% [18]. As a result, normalization was required for comparisons between independent treatments. For normalization, the *gria2α^R^* fraction of treated (morpholinos and cRNA-injected) embryos was normalized to that of the un-injected wild-type embryos.

### Apoptosis assay

Whole mount TUNEL (terminal deoxynucleotidyl transferase-mediated dUTP nick end-labeling) assay were performed using TMR *in situ* death detection kit (Roche), essentially as describe by Cole and Ross [46]. Embryos were dechorionated and fixed in 4% PFA/PBS overnight at 4°C. They were washed in PBS, dehydrated through series of ethanol/PBS and treated with acetone for 10 min at −20°C. After rehydration in PBS, embryos were permeabilized in freshly prepared 0.1% Triton X-100 and 0.1% sodium citrate/PBS solution for 10–15 min at room temperature. Embryos were washed in PBS and incubated with the TMR-labeled nucleotide and terminal deoxynucleotidyl transferase (Roche) for 1 hour at 37°C. Reaction was stopped by rinsing in PBS. The fluorescent signal was visualized and imaged using a confocal microscopy (LSM510, Zeiss). To calculate the fluorescent signals of the epiblast of 5-somite stage embryos, the epiblast region was marked from complied z-stack images (bright field images) before fluorescent intensity was summarized (Image J). Since the enzymatic reaction depended both on the broken DNA ends and the efficiency of reaction, 5 untreated (wild type) embryos of matching stages were always included for internal control. The medium fluorescent intensity of the wild-type epiblast was used to normalize the intensities of the embryos in the same experimental treatment and reaction (relative TUNEL signals). For statistic analysis, unpaired Student's *t-*test single tail was performed (Excel).

### Visualization of brain ventricles

For the images shown in the Figure S2, embryos were dechorionated and stained with Acridine orange before fluorescein dextran injection. Dechorionated embryos were stained by Acridine orange (5 µg/ml) in embryo medium for 10 min as described by Barrallo-Gimeno et al. [47]. Embryos were washed several times until background fluorescence was low (in dark). Embryos were then mounted in low melting agarose. One nl of fluorescein dextran (*Mr* 70,000, Molecular Probes) was injected into the mesencephalic duct and immediately imaged using confocal microscopy (Zeiss LSM510). Images were visualized and captured by LSM510 (Zeiss).

### Lateral line neuromasts labeling

Dechorionated embryos and larvae were soaked in 200 µM 4-(4-diethylaminostyryl)-*N*-methylpyridinium iodide (4-Di-2-Asp, Sigma) for 5 min and rinse [48]. Embryos were visualized and photographed by epifluorescence microscope Zeiss Axioplan 2 and AxioCam HRm.

### Whole-mount Alcian blue cartilage staining

Alcian blue staining was performed as previously described [49]. Embryos were fixed in 4% PFA/PBS at room temperature overnight. PFA of the fixed embryos were washed out by PBST (0.1% Tween 20/PBS) and rinsed with acid alcohol (0.37% HCl in 70% EtOH). Alcian blue (Sigma) in acid alcohol was used to stain the embryos for 4 hours at room temperature. After washing with acid alcohol overnight, the embryos were rehydrated into PBST and treated with 1% trypsin (Sigma) at room temperature for one hour. Embryos were incubated with 4% PFA for twenty minutes and stored in 70% glycerol at 4°C. Images were visualized and captured by Zeiss AxioImager.M1 microscope and Zeiss AxioCam HRc camera.

## Supporting Information

Figure S1 **Quantitative analysis of** ***adar2*** **transcript during embryogenesis.** The amount of *adar2* was determined by comparing to the standard curve and normalized to the amount of *actb1* (relative expression level). Values represented mean ±standard deviation (n = 5). * indicated significant differences (p<0.05) to the 0 hpf by the pair Student's *t* test.
(TIF)

Figure S2 **Brain ventricles are enlarged in the 36-hpf** ***adar2MOt*** **and** ***adar2MOt-p53^AUG^*** **.** Upper panel: lateral view of the 36-hpf head region. Lower panel: dorsal view of the brain ventricles and the distribution of apoptotic cells in the head regions. Red fluorescence shows brain ventricles marked by the injected fluorescein-conjugated dextran and the green fluorescence shows the apoptotic cells stained by Acridine orange. The diencephalic (DiV) and rhombencephalic (RhV) ventricles are enlarged in the morphants. Scale bar represents 100 µm.
(TIF)

Table S1 **Primer sequences for real-time PCR analysis.**
(DOCX)
