# Supplementary figures and images for: Zebrafish Adar2 Edits the Q/R Site of AMPA Receptor Subunit gria2α Transcript to Ensure Normal Development of Nervous System and Cranial Neural Crest Cells
Source: PLoS One. 2014 May 12;9(5):e97133. doi: 10.1371/journal.pone.0097133 (PMC4018279; doi:10.1371/journal.pone.0097133)

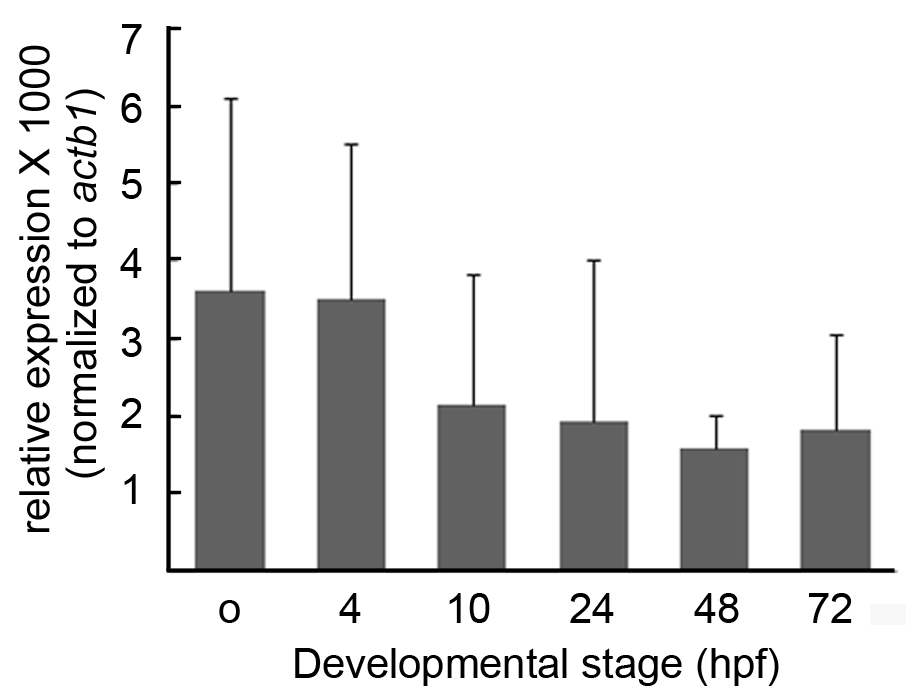

Supplement: Figure S1 — Quantitative analysis of adar2 transcript during embryogenesis. The amount of adar2 was determined by comparing to the standard curve and normalized to the amount of actb1 (relative expression level). Values represented mean ±standard deviation (n = 5). * indicated significant differences (p<0.05) to the 0 hpf by the pair Student's t test. (TIF) [file pone.0097133.s001.tif]

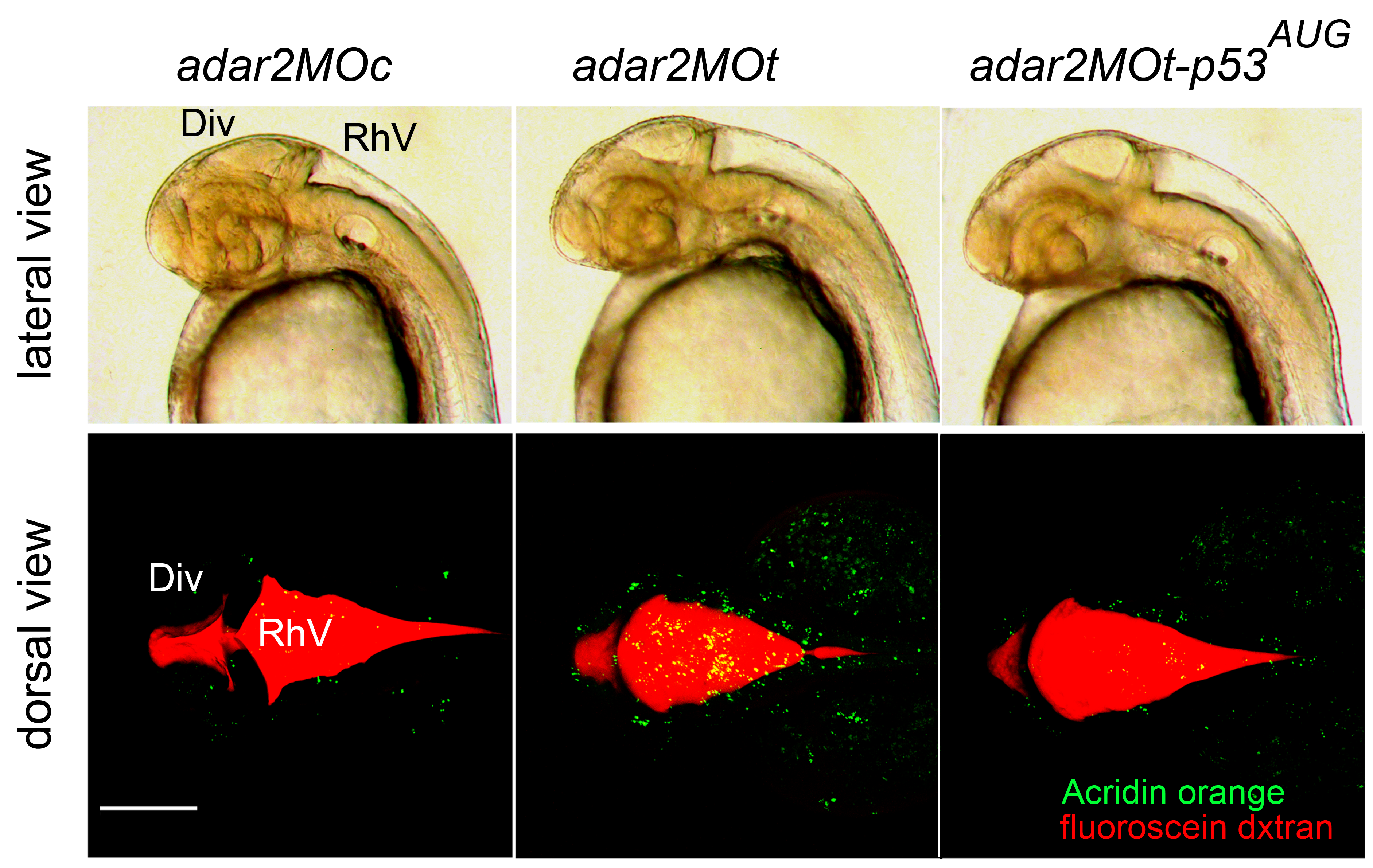

Supplement: Figure S2 — Brain ventricles are enlarged in the 36-hpf adar2MOt and adar2MOt-p53AUG . Upper panel: lateral view of the 36-hpf head region. Lower panel: dorsal view of the brain ventricles and the distribution of apoptotic cells in the head regions. Red fluorescence shows brain ventricles marked by the injected fluorescein-conjugated dextran and the green fluorescence shows the apoptotic cells stained by Acridine orange. The diencephalic (DiV) and rhombencephalic (RhV) ventricles are enlarged in the morphants. Scale bar represents 100 µm. (TIF) [file pone.0097133.s002.tif]
