# Supplementary material for: Zebrafish Adar2 Edits the Q/R Site of AMPA Receptor Subunit gria2α Transcript to Ensure Normal Development of Nervous System and Cranial Neural Crest Cells
Source: PLoS One. 2014 May 12;9(5):e97133. doi: 10.1371/journal.pone.0097133 (PMC4018279; doi:10.1371/journal.pone.0097133)
Supplement: Table S1 — Primer sequences for real-time PCR analysis. (DOCX) [file pone.0097133.s003.docx]

**Supporting information**

Table S1. Primer sequences for real-time PCR analysis.

| Gene | accession number [ref] | sequence (5’ to 3’) |
| --- | --- | --- |
| *actb1* | NM_131031.1 | CTCCATCATGAAGTGCGACGT |
|  | [16] | CAGACGGAGTATTTGCGCTCA |
| *adar1* | NM_131596.1 | TGATAAGATCCTGCGCTGGAA |
|  |  | TTGGACAATCGGCAGCACA |
| *adar2* | NM_131610 | TATATCAGCACGTCGCCCTGT |
|  |  | TTGGTTCGTAGTTGTCCACGG |
| *gria1α* | XM_005157305 | CAGCGCAAACCCTGCGACA |
|  | [16] | CATGGCCAGACCCAGACC |
| *gria2α* | XM_005170897 | TCGAAAGTGCTGAAGAACTGG |
|  | [16] | CGCTCTTCATGTACTGCCACA |
| *ccng1* | XM_005173092.1 | TGTTCATTTGGATCGGTGCA |
|  |  | TCAAAACCCTTCTCCCTCCTC |
| *neuroD* | NM_130978.1 | CAGACTGCACCAGTCCTCATTT |
|  |  | TCGAATTCCGAAGAAGGCTCGT |
| *neurog1* | NM_131041.1 | GCACAACCTTAACGACGCATT |
|  |  | CCCAGATGTAGTTGTGAGCGAA |
| *mdm2* | XM_005164845.1 | CCGACAGCTTTCCCTGACAT |
|  |  | CCGAGTTGCTGTTTGCATCC |
| 3'-*p53* | XM_005165103.1 | ATGAGGAGATCTTTACCCTGCAG |
|  |  | TGAGGCAGGCACCACATC |
| *Δ113p53* | XM_005165103.1 | ATATCCTGGCGAACATTTGGAG |
|  | [21] | CAACGTCCACCACCATTTGA |
| *sox9a* | NM_131643.1 | AGTACCCGCACCTCCACAAC |
|  |  | CACGAAGGGACGCTTTTCC |
| *sox9b* | NM_131644.1 | ACTCTGGAGACTGCTGAACGA |
|  |  | TGAGCCGCTCTTCACTGATT |
